# Molecular-Level Insights into Meta-Phenylenediamine and Sulfonated Zinc Phthalocyanine Interactions for Enhanced Polyamide Membranes: A DFT and TD-DFT Study

**DOI:** 10.3390/polym17152019

**Published:** 2025-07-24

**Authors:** Ameni Gargouri, Bassem Jamoussi

**Affiliations:** 1 Mathematics Department, College of Sciences and Humanities, Prince Sattam bin Abdulaziz University, Al-Kharj 11912, Saudi Arabia; 2 Department of Environment, Faculty of Environmental Sciences, King Abdulaziz University, Jeddah 21589, Saudi Arabia; bissuomaj@kau.edu.sa

**Keywords:** phthalocyanine–MPD interaction, density functional theory (DFT), dihedral rotation, thin-film composite membranes, antifouling water treatment membranes

## Abstract

Access to clean water is a pressing global concern and membrane technologies play a vital role in addressing this challenge. Thin-film composite membranes prepared via interfacial polymerization (IPol) using meta-phenylenediamine (MPD) and trimesoyl chloride (TMC) exhibit excellent separation performance, but face limitations such as fouling and low hydrophilicity. This study investigated the interaction between MPD and sulfonated zinc phthalocyanine, Zn(SO_2_^−^)_4_Pc, as a potential strategy for enhancing membrane properties. Using Density Functional Theory (DFT) and Time-Dependent DFT (TD-DFT), we analyzed the optimized geometries, electronic structures, UV–Vis absorption spectra, FT-IR vibrational spectra, and molecular electrostatic potentials of MPD, Zn(SO_2_^−^)_4_Pc, and their complexes. The results show that MPD/Zn(SO_2_^−^)_4_Pc exhibits reduced HOMO-LUMO energy gaps and enhanced charge delocalization, particularly in aqueous environments, indicating improved stability and reactivity. Spectroscopic features confirmed strong interactions via hydrogen bonding and π–π stacking, suggesting that Zn(SO_2_^−^)_4_Pc can act as a co-monomer or additive during IPol to improve polyamide membrane functionality. A conformational analysis of MPD/Zn(SO_2_^−^)_4_Pc was conducted using density functional theory (DFT) to evaluate the impact of dihedral rotation on molecular stability. The 120° conformation was identified as the most stable, due to favorable π–π interactions and intramolecular hydrogen bonding. These findings offer computational evidence for the design of high-performance membranes with enhanced antifouling, selectivity, and structural integrity for sustainable water treatment applications.

## 1. Introduction

The Meta-phenylenediamine (MPD) is a widely employed monomer in interfacial polymerization (IPol) processes for fabricating thin-film composite (TFC) membranes, particularly in ultrafiltration (UF) and reverse osmosis (RO) technologies. When reacted with trimesoyl chloride (TMC), MPD forms a highly cross-linked polyamide layer essential for membrane selectivity in water desalination and purification [1,2]. However, membranes derived from MPD often suffer from issues such as hydrophobicity, chlorine-induced degradation, and biofouling [3]. To address these challenges, numerous strategies—such as nanomaterial incorporation, organic functionalization, and polymer blending—have been developed to enhance membrane permeability, mechanical robustness, fouling resistance, and improve overall membrane performance [4,5].

Among the most promising modifications is the integration of phthalocyanines (Pcs) and their metal complexes (MPcs), which exhibit strong visible light absorption, high chemical stability, and efficient charge transport. Specifically, zinc phthalocyanine (ZnPc) derivatives have garnered attention for applications in optoelectronics, gas sensors, catalysis, and photodynamic antimicrobial therapy [6,7,8]. Functionalization of ZnPc with sulfonate groups (-SO_2_^−^) further improves solubility and enhances reactivity in polar environments [9,10]. In contrast to previously reported nanomaterials (e.g., graphene oxide, silica nanoparticles [11] and functionalized monomers (e.g., amine-terminated PEGs or sulfonated diamines [12,13], which often act as passive additives or are physically dispersed in the polyamide matrix, sulfonated ZnPc offers a multifunctional and structurally integrated approach. Its π-conjugated macrocyclic architecture enables direct co-facial interactions with MPD through π–π stacking [14], while the presence of multiple sulfonate groups promotes hydrogen bonding and electrostatic interactions during interfacial polymerization [15]. This dual-interaction mechanism facilitates more uniform incorporation within the polyamide network, minimizing aggregation and improving membrane homogeneity [16].

Despite progress in MPD/TMC-based membranes, the molecular-level interaction between MPD and sulfonated ZnPc derivatives in the aqueous phase—and their cooperative behavior during IPol—remains underexplored. Computational approaches such as Density Functional Theory (DFT) and Time-Dependent DFT (TD-DFT) offer a powerful means to investigate these interactions and predict membrane performance. These methods allow for detailed assessment of electronic structure, molecular orbital distributions, vibrational dynamics, and charge localization under solvent effects [17,18].

Recent advances in computational chemistry have enabled the use of Density Functional Theory (DFT) and Time-Dependent DFT (TD-DFT) to extensively investigate the electronic structure, spectroscopic properties, and reactivity of phthalocyanine-based systems [19,20]. These methods are particularly effective in analyzing molecular orbital distributions, vibrational dynamics, and charge localization—essential parameters for optimizing the functional performance of membrane additives. Building upon the findings of Jamoussi et al. [21], who demonstrated the potential of ZnPc-based platforms for enhancing anti-biofouling performance in water treatment membranes using DFT and molecular docking approaches, the present study focuses on the structural and electronic properties of sulfonated zinc phthalocyanine, Zn(SO_2_^−^)_4_Pc, and its interaction with meta-phenylenediamine (MPD). Employing DFT and TD-DFT methods with the 6-31G(d) basis set, we examine UV–Vis spectral features—particularly π → π* transitions such as the Q- and B-bands—along with FT-IR vibrational profiling to characterize bond stretching and functional group behavior, and Molecular Electrostatic Potential (MEP) mapping to visualize reactive regions within the complexes. Furthermore, we investigate the influence of solvation effects in water on the electronic structure of MPD/Zn(SO_2_^−^)_4_Pc complexes, providing insight into their potential for improving membrane selectivity, antifouling behavior, and chemical robustness during interfacial polymerization processes. The structural and electronic properties of MPD-linked phthalocyanine complexes are influenced by their conformational flexibilities [22,23]. Understanding how the dihedral angle between the MPD linker and phthalocyanine core affects molecular stability is necessary for optimizing these hybrid systems in membrane and optoelectronic applications. To address this, we performed a systematic dihedral scan of MPD/Zn(SO_2_^−^)_4_Pc using Density Functional Theory (DFT) to investigate the relationship between conformation and stability.

This study provides molecular-level insights into Zn(SO_2_^−^)_4_Pc’s suitability as a co-functional monomer in interfacial polymerization, contributing to the development of high-performance polyamide membranes with improved stability, selectivity, and antifouling characteristics in water treatment and separation applications.

## 2. Materials and Methods

### 2.1. Materials and Computational Environment

All simulations and computational analyses were performed using the Gaussian 09 software suite (Revision A02; Gaussian Inc., Wallingford, CT, USA). The visualization of optimized structures, vibrational modes, and electrostatic potential surfaces was carried out using GaussView 6.0 [24]. Calculations were executed on the Aziz High-Performance Computing Cluster hosted by King Abdulaziz University’s HPC center.

The molecular systems investigated include Zn(SO_2_^−^)_4_Pc, MPD, and the MPD/Zn(SO_2_^−^)_4_Pc complex in both gas and aqueous phases. These molecular systems were constructed using standard geometries, and all atomic parameters used were derived from established quantum chemistry libraries.

### 2.2. Geometry Optimization

All molecular geometries were fully optimized without symmetry constraints using the B3LYP functional, which is widely employed in the literature for phthalocyanine systems. A hybrid basis set was adopted: LANL2DZ pseudopotential was used for Zn, and the 6-31G(d) basis set was applied to H, C, N, O, and S atoms [25,26]. Frequency calculations were carried out to confirm that all optimized structures correspond to true energy minima, i.e., with no imaginary vibrational frequencies [27]. For the Zn(SO_2_^−^)_4_Pc, two Density Functional Theory (DFT) methodologies were employed: one utilizing the B3LYP functional without dispersion correction, and the other incorporating Grimme’s D3BJ empirical dispersion correction. This comparative study was undertaken to evaluate the impact of dispersion interactions on the structural stability, optimized geometry, and electronic properties of the isolated molecule.

### 2.3. TD-DFT and UV–Vis Spectral Simulation

Time-Dependent Density Functional Theory (TD-DFT) has become the most widely used theoretical approach for simulating the optical properties of organic and inorganic molecules [28]. In this contribution, TD-DFT was used to simulate the UV–Vis absorption spectra of Zn(SO_2_^−^)_4_Pc. Calculations were conducted at the B3LYP/6-31G(d) level. Transition energies and oscillator strengths were analyzed to identify characteristic electronic transitions, particularly the Q-band and B-band regions, which are relevant to optoelectronic applications.

### 2.4. FT-IR Vibrational Analysis

Vibrational spectra were simulated using the B3LYP/6-31G(d) level of theory for Zn(SO_2_^−^)_4_Pc. A frequency scaling factor of 0.9614 was applied to correct computed wavenumbers, aligning them more closely with experimentally observed FT-IR spectra for phthalocyanine derivatives. This analysis identified stretching modes associated with Zn–N, C–N, C–C, and SO_2_^−^ functional groups.

### 2.5. Solvent Effects Using PCM

To explore the impact of solvation, single-point energy calculations were performed using the Polarizable Continuum Model (PCM) with water as the solvent. These calculations assessed energy stabilization and dipole moment variation in aqueous conditions, which are critical for membrane and catalytic applications.

### 2.6. Frontier Molecular Orbitals and Global Reactivity

The HOMO–LUMO energy gaps and global reactivity descriptors (ionization potential, electron affinity, chemical hardness, electrophilicity index) were determined via Koopman’s theorem for closed-shell systems. These values were used to evaluate the reactivity and stability of the MPD/Zn(SO_2_^−^)_4_Pc system under different phases. Molecular electrostatic potential (MEP) analysis was performed to identify electrophilic and nucleophilic reactive sites, which provided insight into possible intermolecular interactions and functionalization sites [29].

### 2.7. Conformational Analysis via Dihedral Scan

To examine the conformational preferences of the MPD/Zn(SO_2_^−^)_4_Pc hybrid, we performed relaxed geometry optimizations at selected dihedral angles (60°, 90°, 120°, and 180°) using DFT at the B3LYP/6-31G(d) level of theory in the gas phase [30]. The dihedral angle is defined by atoms 28 (Pc ring carbon anchor), 32 (linker carbon), 66 (MPD phenyl carbon), and 67 (adjacent MPD ring carbon). Each structure was assigned a −4 charge and a singlet multiplicity. The optimizations were confirmed as true minima by the absence of imaginary vibrational frequencies.

## 3. Results

### 3.1. Geometry of Molecular Optimization of the MPD, Zn(SO_2_^−^)_4_Pc and MPD/Zn(SO_2_^−^)_4_Pc

**Figure 1:**
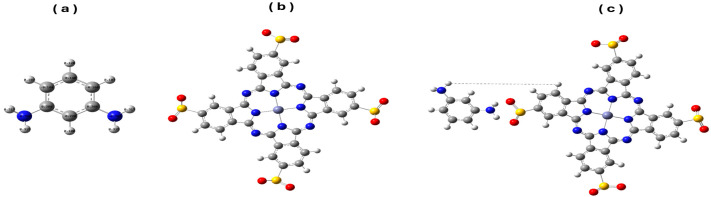
Model molecules for MPD (**a**), Zn(SO_2_^−^)_4_Pc (**b**) and MPD/Zn(SO_2_^−^)_4_Pc (**c**) calculated at B3LYP/LANL2DZ level. [C in grey, H in white, O in red, N in blue, Zn in purple and S in yellow].

The minimum total energies of the MPD, Zn(SO_2_^−^)_4_Pc and MPD/Zn(SO_2_^−^)_4_Pc compounds were determined by optimizing the respective molecules using the Gaussian 09 W software and the B3LYP functional with the 6-31G(d) basis set. Among these, the Zn(SO_2_^−^)_4_Pc complex was further optimized using two methodologies: B3LYP without dispersion correction and B3LYP with Grimme’s D3BJ empirical dispersion correction, to evaluate the impact of dispersion interactions on its structural and electronic properties. The optimized geometries of the MPD, Zn(SO_2_^−^)_4_Pc, MPD/Zn (SO_2_^−^)_4_Pc molecules are presented in Figure 1.

For Zn(SO_2_^−^)_4_Pc (Figure 1b) and MPD/Zn(SO_2_^−^)_4_Pc (Figure 1c), the LANL2DZ pseudopotential is applied to the Zn atom. Specific bond constraints were imposed on the sulfinate groups to preserve essential structural features during the computational: S=O bonds were fixed at 1.40 Å, and S–O bonds were fixed at 1.60 Å, ensuring an accurate representation of the geometric constraints. The interaction between MPD and Zn(SO_2_^−^)_4_Pc offers essential insights into the behavior of the MPD/Zn(SO_2_^−^)_4_Pc copolymer, which plays a key role in the interfacial polymerization with TMC to form polyamides. As indicated in Figure 1, optimization of the model system MPD/Zn(SO_2_^−^)_4_Pc showed that the origin of the interaction was mainly due to the formation of hydrogen bonding and π–π stacking. The amino groups in MPD (-NH_2_) possibly formed hydrogen bonds with the oxygen atoms in the sulfinyl groups (SO_2_^−^) of Zn(SO_2_^−^)_4_Pc, stabilizing the complex. Additionally, the aromatic rings of MPD and Zn(SO_2_^−^)_4_Pc core imply π–π interactions further contribute to the stability of the system, supporting the hypothesis that the MPD monomer can interact with the TMC monomer to form polyamide chains during interfacial polymerization as a co-polymer-with-the-functional group Zn(SO_2_^−^)_4_Pc.

To evaluate the influence of dispersion forces on the gas-phase structure of Zn(SO_2_^−^)_4_Pc, we conducted a comparative analysis using B3LYP and B3LYP-GD3BJ functionals. The B3LYP-GD3BJ approach resulted in a more stabilized structure, evidenced by a notably lower total electronic energy (−5638.428473 Hartree) in comparison to the B3LYP-only outcome (−5638.225264 Hartree), indicating a stabilization of approximately 127.4 kcal/mol. Additionally, the dipole moment exhibited a slight increase (from 2.55 D to 2.59 D), signifying subtle alterations in the charge distribution. Although the HOMO–LUMO gap remained nearly constant (1.32 eV vs. 1.31 eV), the optimization process using GD3BJ necessitated a significantly greater number of steps (225 vs. 20), suggesting a more complex potential energy surface when dispersion is considered. Although dispersion correction improves the energy description in the gas phase, in the case of the Zn(SO_2_^−^)_4_Pc/water complex, the use of B3LYP without dispersion correction is justified. The interactions in this solvated system are primarily governed by electrostatic and hydrogen bonding, which are accurately captured by the B3LYP functional. In addition, the implicit solvation model (e.g., PCM) reduces the importance of long-range dispersion forces by screening them in a polar solvent environment. Employing dispersion corrections in such settings introduces unnecessary computational complexity without significantly altering the electronic structure, as evidenced by its minimal impact on the HOMO–LUMO gap. Therefore, the use of B3LYP without GD3 offers a reliable and computationally efficient approach for studying highly charged polar systems, such as Zn(SO_2_^−^)_4_Pc in aqueous media.

Table 1 lists all the minimum total energies of MPD, Zn(SO_2_^−^)_4_Pc and MPD/Zn(SO_2_^−^)_4_Pc with their respective dipole moments.

Table 1 provides a comparison of the minimum total energy (in Hartree) and dipole moments (in Debye) for MPD, Zn(SO_2_^−^)_4_Pc, and their interactions (MPD/Zn(SO_2_^−^)_4_Pc) in the gas phase and in water. The total energy values reflect the stability of each state, with a lower energy corresponding to a higher stability. For MPD in the gas phase, the total energy is −342.954877 Hartree, which is the least negative because of its smaller size and molecular simplicity. In contrast, Zn(SO_2_^−^)_4_Pc in the gas phase exhibits a significantly lower energy of −5638.225264 Hartree, indicating higher stability due to its greater molecular complexity and the stabilizing contributions of zinc and sulfonyl groups. When MPD interacts with Zn(SO_2_^−^)_4_Pc to form the MPD/Zn(SO_2_)_4_Pc complex in the gas phase, the energy further decreases to −5981.218660 Hartree, demonstrating strong interactions and enhanced stability. In water, the MPD/Zn(SO_2_^−^)_4_Pc complex is slightly more stable, with a total energy of −5981.82393 Hartree, highlighting the stabilizing effects of solvation. The minor energy difference between the gas phase and water (~0.605 Hartree, or ~381.5 kcal/mol) indicates plays a significant role in stabilizing polar groups in the complex.

Dipole moment values reflect the polarity and electronic distribution of each molecule or complex. MPD in the gas phase has a moderate dipole moment of 2.603122 Debye, attributed to its symmetrical structure with polar amino groups. Zn(SO_2_^−^)_4_Pc exhibits a slightly lower dipole moment of 2.554394 Debye, suggesting more delocalized electron distributions. Upon MPD/Zn(SO_2_^−^)_4_Pc complex formation, the dipole moment increases significantly to 19.817231 Debye in the gas phase, reflecting enhanced charge separation and strong intermolecular interactions. In water, the dipole moment increases further to 23.18453 Debye due to the polarizing effect of the solvent environment, which amplifies charge separation across the complex.

The interaction occurring in water influences the electronic properties of the system. The calculated solvation energy of the complex was calculated using Equation (1), which gives the value ΔE_solvation_ ≈ 381.47 kcal/mol.

ΔE_solvation_ = E_gas_ − E _water_ (1)

The calculated solvation energy indicated that water significantly stabilized the interaction between MPD and Zn(SO_2_^−^)_4_Pc, thereby improving the overall binding strength and enhancing the suitability of the system for aqueous-phase applications.

These results emphasize the potential of the MPD/Zn(SO_2_^−^)_4_Pc complex as a highly polar system suitable for interfacial polymerization, which requires strong polarity and stability. To further explore how geometry influences the stability and electronic properties of the MPD/Zn(SO_2_^−^)_4_Pc complex, we examined the effect of dihedral rotation between the MPD moiety and the phthalocyanine core.

### 3.2. Conformational Energy and Electronic Structure of MPD/Zn(SO_2_^−^)_4_Pc

#### 3.2.1. Conformational Energy and Stability

The calculated electronic energies revealed subtle but meaningful differences in the stabilities of the conformers (Table 2). The 120° conformation exhibited the lowest electronic energy (−5981.218660 Hartree), indicating that it was the most thermodynamically stable. This stability is closely followed by the 180° and 60° conformers, whereas the 90° conformation is slightly less favorable.

**Figure 2:**
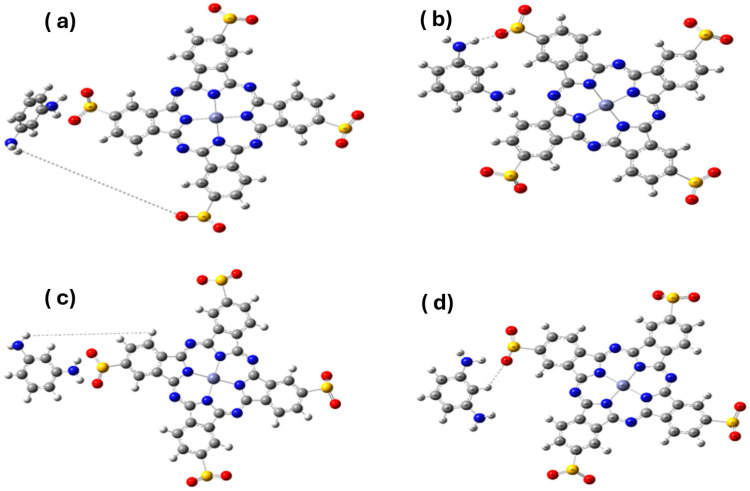
Optimized conformers of the MPD/Zn(SO_2_^−^)_4_Pc system at dihedral angles of (**a**) 60°, (**b**) 90°, (**c**) 120°, and (**d**) 180°.

The enhanced stability at 120° can be attributed to favorable π–π interactions between the MPD phenyl ring and the peripheral aromatic rings of phthalocyanine, as well as the formation of intramolecular hydrogen bonds between the MPD amine groups and sulfonate oxygen atoms (Figure 2). These interactions occur without distorting the planarity of the phthalocyanine core, thus preserving its extended π-conjugation. In contrast, the 60° conformation induces steric strain, while the 90° angle disrupts favorable orbital overlap due to perpendicular alignment. At 180°, the MPD was spatially separated, reducing cooperative interactions.

#### 3.2.2. Effect on Electronic Structure: HOMO–LUMO Gap

Frontier molecular orbital (FMO) analysis showed that the HOMO–LUMO gap was modestly influenced by dihedral rotation (Table 3). The energy gaps ranged from 1.2089 to 1.2345 eV, with the largest gap observed at 180°, followed by 120°. The 90° conformation exhibited the smallest gap, likely due to the reduced π-conjugation and orbital delocalization caused by geometric misalignment.

The findings indicate that conformational tuning can subtly influence the electronic properties of the hybrid, which may be pertinent for optimizing charge transport or light-absorption characteristics in applied systems.

### 3.3. Analysis and Comparison of HOMO-LUMO Gap Energies

**Figure 3:**
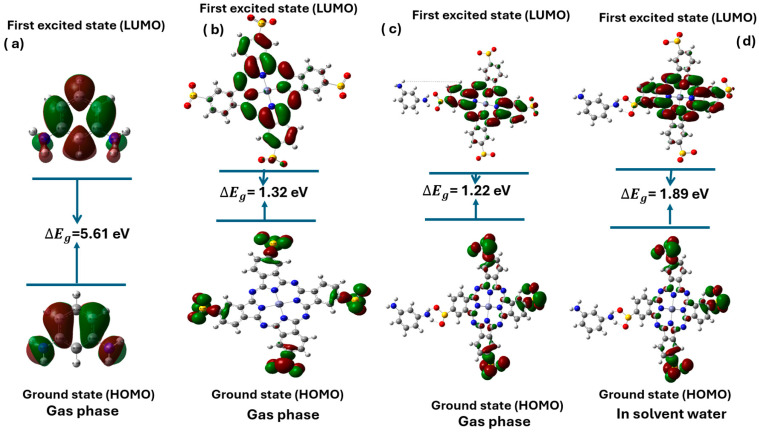
HOMO-LUMO gap of MPD (**a**), Zn (SO2^−^) _4_Pc (**b**), MPD/Zn(SO_2_^−^)_4_Pc (**c**) (gas phase) and MPD/Zn(SO2^−^)_4_Pc (**d**) (solvent water).

Figure 3 presents the HOMO-LUMO energy gaps ΔEg for different molecular systems, including MPD, Zn(SO_2_^−^)_4_Pc, and their complexes in the gas and aqueous phases.

The energy gap values indicate electronic properties such as conductivity and charge transfer efficiency. The MPD molecule in the gas phase exhibited the largest energy gap ΔEg = 5.61 eV, suggesting that it is an electrical insulator with minimal electron delocalization, making it unsuitable for conductive applications. In contrast, Zn(SO_2_^−^)_4_Pc in the gas phase showed a significantly lower energy gap ΔEg = 1.32 eV, indicating improved electronic conductivity and potential as a semiconductor owing to its extended conjugation and charge delocalization (Table 3).

When MPD interacts with Zn(SO_2_^−^)_4_Pc in the gas phase, the energy gap further decreases to 1.22 eV, highlighting a strong charge transfer interaction between the two molecules. This bandgap reduction suggests that the MPD/Zn(SO_2_^−^)_4_Pc complex has enhanced optoelectronic properties compared to its individual components. However, when the same complex was solvated in water, the energy gap increases to 1.89 eV. This change indicates that solvent stabilization affects the electronic structure. The increase in the energy gap implies that solvation reduces the charge transfer efficiency due to the polarization effects and interactions with water molecules. Additionally, the polarity of water and its ability to form hydrogen bonds contribute to the stabilization of energy levels in MPD/Zn(SO_2_^−^)_4_Pc. The hydrogen bonding between the water molecules and the MPD/Zn(SO_2_^−^)_4_Pc complex leads to an increase in the energy gap [31]. This effect can be attributed to solvation dynamics, specific solute–solvent interactions, and environmental electronic effects. While this suggests a slight decrease in electronic reactivity in aqueous environments, it does not necessarily compromise the membrane performance. The moderate bandgap (1.89 eV) in water remains within the range suitable for optoelectronic and photodynamic applications. Additionally, this stabilization can enhance chemical robustness and minimize undesirable side reactions in aqueous conditions, which is beneficial for membrane stability. Thus, the observed increase in the energy gap is not detrimental; rather, it reflects a balanced electronic profile suitable for operation in aqueous media, particularly in photocatalytic or light-responsive membrane systems.

### 3.4. Global Reactivity Descriptors

Using Koopman’s theorem for closed-shell molecules, key parameters such as electron affinity (A), ionization potential (I), chemical hardness (η), chemical softness (S), electronic chemical potential (μ), and global electrophilicity index (ω) were calculated for MPD, Zn(SO_2_^−^)_4_Pc, and MPD/Zn(SO_2_^−^)_4_Pc, as summarized in Table 4 [32,33,34,35].

The analysis of global chemical reactivity descriptors provided valuable insights into the electronic properties and reactivity of the studied molecules (Table 5). The HOMO (highest occupied molecular orbital) and LUMO (lowest unoccupied molecular orbital) energies reveal that MPD has the lowest E_HOMO (−0.18457 Hartree), indicating it is the best electron donor, while Zn (SO_2_^−^)_4_Pc has the highest E_LUMO (0.17139 Hartree), making it the strongest electron acceptor. Notably, MPD/Zn(SO_2_^−^)_4_Pc in water has a positive E_LUMO (0.09829 Hartree), suggesting a reduced electron-accepting ability in aqueous environments. The ionization potential (IP), which represents a molecule’s resistance to electron loss, is highest for MPD (0.18457 Hartree), confirming its stability. Meanwhile, the electron affinity (EA) is highest for Zn(SO_2_^−^)_4_Pc (−0.17139 Hartree), reinforcing its strong electron-accepting character.

In terms of chemical hardness (η) and softness (s), MPD is the hardest molecule (0.103075 Hartree), making it the most stable and least reactive, while Zn(SO_2_^−^)_4_Pc is the softest (0.02421 Hartree), indicating high reactivity. Soft molecules tend to be more chemically interactive, which explains why Zn(SO_2_^−^)_4_Pc exhibits a high softness value (20.64835). The chemical potential (μ), which governs the tendency of a molecule to exchange electrons, is highest for Zn(SO_2_^−^)_4_Pc (0.147175 Hartree), making it the most electron-releasing species, whereas MPD/Zn(SO_2_^−^)_4_Pc in water has the lowest μ (−0.133505 Hartree) indicating that it attracts electrons more effectively in aqueous solutions. The electronegativity (χ) values indicate that MPD/Zn(SO_2_^−^)_4_Pc in water has the highest electronegativity (0.517355 Hartree), making it the strongest electron-attracting species, whereas Zn(SO_2_^−^)_4_Pc has the lowest (0.147175 Hartree), suggesting a weaker electron-withdrawing ability. The electrophilicity index (ω) further confirms that Zn(SO_2_^−^)_4_Pc is the most electrophilic species (0.447253 Hartree), making it highly susceptible to electron transfer, whereas MPD is the least electrophilic (0.032216 Hartree), highlighting its stability.

### 3.5. Molecular Electrostatic Potential (MEP) Analysis

Molecular Electrostatic Potential (MEP) is crucial for identifying electrophilic and nucleophilic sites within a compound [36]. The maximum positive region, depicted in blue, indicates areas most susceptible to nucleophilic attack, whereas the negative region, shown in red, represents sites preferentially targeted by electrophiles [37]. Molecular Electrostatic Potential (MEP) analysis of Zn (SO_2_^−^)_4_Pc and MPD/Zn(SO_2_^−^)_4_Pc in different environments revealed significant differences in charge distribution, reactivity, and stability (Figure 3). In the gas phase, Zn(SO_2_^−^)_4_Pc exhibits highly localized charge regions, with strong negative electrostatic potential (red) around the sulfonate (-SO_2_^−^) groups, making them prone to electrophilic interactions, while the positive electrostatic potential (blue) is concentrated near the Zn^2+^ metal center, making it attractive to nucleophiles. This strong charge separation suggests high reactivity and potential applications in catalysis and charge transfer processes.

In contrast, the MPD/Zn(SO_2_^−^)_4_Pc system in the gas phase showed a more homogeneous charge distribution, with less localized electrostatic potential variations, indicating increased electronic delocalization and stability. The presence of MPD (meta-phenylenediamine) bridges reduces charge localization, enhancing conductivity while lowering molecular reactivity compared to standalone Zn(SO_2_^−^)_4_Pc. When solvated in water, MPD/Zn(SO_2_^−^)_4_Pc experiences further electrostatic potential diffusion as solvent interactions shield and stabilize charge separation, reducing the reactivity of the molecule. The solvent-induced charge screening effect reduces the extreme positive and negative regions, indicating stronger solvation effects that enhance stability and solubility. Comparatively, Zn(SO_2_^−^)_4_Pc in the gas phase was the most reactive, favoring strong charge-transfer interactions, whereas MPD/Zn(SO_2_^−^)_4_Pc in the gas phase was more stable with a delocalized electronic structure. The aqueous-phase MPD/Zn(SO_2_^−^)_4_Pc is the least reactive but most stable, making it more suitable for applications requiring enhanced solubility and stability in polar environments, such as aqueous-phase catalysis, sensing, and electronic materials. These findings highlight the impact of functionalization and solvent effects on the electronic properties and the potential applications of Zn(SO_2_^−^)_4_Pc-based systems.

### 3.6. Analysis of the UV–Vis Spectrum of Zn(SO_2_^−^)_4_Pc Using TD-DFT at the 6-31G(d) Basis Set

**Figure 4:**
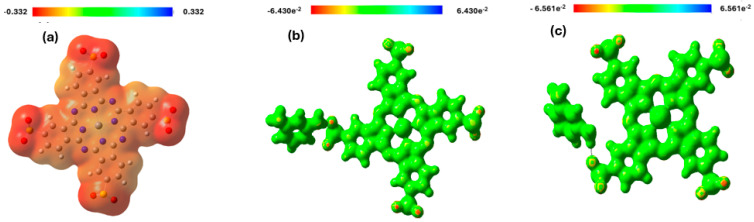
Molecular Electrostatic Potential map of (**a**) Zn (SO_2_^−^)_4_Pc (gas phase), (**b**) MPD/Zn(SO_2_^−^)_4_Pc (gas phase) and (**c**) MPD/Zn(SO_2_^−^)_4_Pc (**c**) (in water).

The UV–Vis spectrum of Zn(SO_2_^−^)_4_Pc in the gas phase (Figure 4), computed using Time-Dependent Density Functional Theory (TD-DFT) on the 6-31G(d) basis set, revealed significant electronic transitions that provided insights into the optical properties of the molecule. The spectrum exhibits a strong absorption peak at 611.417 nm with an oscillator strength of 0.0796, indicating a significant π → π* electronic transition, which is characteristic of phthalocyanine-based systems. This dominant peak corresponds to the Q-band, a well-known feature of phthalocyanines (Pcs) and metallo-phthalocyanines (MPcs), arising from the HOMO → LUMO excitations within the π-conjugated macrocyclic system. Additionally, weaker absorptions were observed in the higher-energy region (300–500 nm), corresponding to B-band (Soret band) transitions, which involve higher-energy π → π* excitations. The calculated Q-band at 611 nm is consistent with experimental values reported for sulfonated Zn-phthalocyanines, which typically range between 600 and 700 nm [38], thus validating our TD-DFT approach.

The presence of Zn^2+^ at the core influences the electronic distribution and slightly shifts the absorption band compared with metal-free phthalocyanines. Additionally, the sulfonate (-SO_2_^−^) groups likely contributed to spectral broadening and minor shifts, affecting the electronic structure and solubility properties of the molecule. The predicted absorption values align well with the experimental ZnPc complexes, which typically absorb in the 600–700 nm range, confirming the reliability of the TD-DFT method. The moderate oscillator strength (0.0796) suggests a reasonable transition probability, which is characteristic of Zn phthalocyanines.

The strong Q-band absorption makes Zn(SO_2_^−^)_4_Pc a promising candidate for application in photodynamic therapy (PDT), organic photovoltaics (OPVs), and optical sensors. Additionally, its broad spectral response suggests its potential for use in light-harvesting materials and nonlinear optical devices. Overall, the TD-DFT-computed spectrum highlights the significant electronic conjugation in Zn(SO_2_^−^)_4_Pc, making it suitable for optoelectronic and photonic applications.

### 3.7. Analysis of the FT-IR Spectrum and Vibrational Modes of Zn(SO_2_^−^)_4_Pc in the Gas Phase Using RB3LYP/6-31G(d)

**Figure 5:**
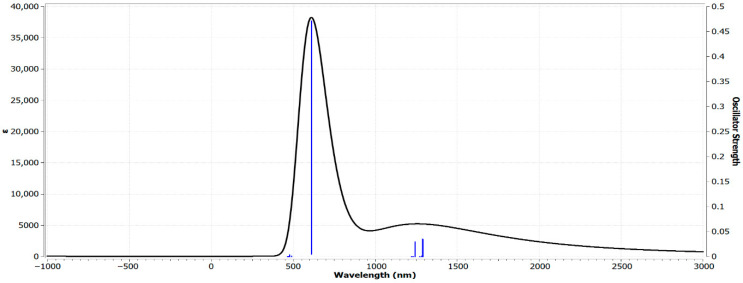
UV–Vis spectrum of Zn(SO_2_^−^)_4_Pc in the gas phase, computed using TD-DFT on the 6-31G(d) basis set.

The Fourier Transform Infrared (FT-IR) spectrum of Zn(SO_2_^−^)_4_Pc in the gas phase, computed using the RB3LYP functional with the 6-31G(d) basis set, provided valuable insights into the molecular vibrational modes and functional group interactions (Figure 5). The spectrum reveals multiple absorption peaks that correspond to different bond stretching and bending modes, reflecting the structural characteristics of Zn-phthalocyanine (ZnPc) with sulfonate (-SO_2_^−^) groups. In the low-frequency region (400–1000 cm^−1^), strong peaks are attributed to Zn-N stretching and Zn-N-C bending vibrations, which are characteristic of metallo-phthalocyanines and play crucial roles in defining the electronic and coordination properties of the molecule. The 1000–1300 cm^−1^ region exhibits intense peaks associated with C-N and C-C stretching modes, indicative of the π-conjugated electronic system that influences the optical and electronic behavior of the molecule. The sulfonate (-SO_2_^−^) groups exhibit distinct asymmetric and symmetric stretching vibrations between 1100 and 1350 cm^−1^, confirming their strong electronic interactions with the ZnPc core. Additionally, the sharp peak near 950 cm^−1^ corresponds to out-of-plane deformations of the SO_2_^−^ groups, which influence the structural stability and solubility of the molecule. The high-frequency region (~3000 cm^−1^) shows weak C-H stretching vibrations, consistent with the aromatic nature and electron delocalization within the macrocyclic system.

**Figure 6:**
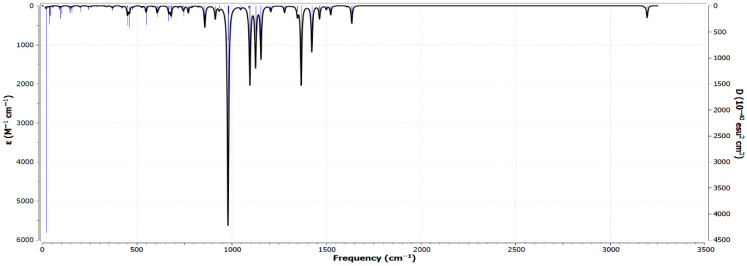
Fourier Transform Infrared (FT-IR) spectrum of Zn(SO_2_^−^)_4_Pc in the gas phase, computed using the RB3LYP functional with the 6-31G(d) basis set.

The computed FT-IR spectrum (Figure 6) aligns well with experimental trends observed in metallo-phthalocyanine derivatives, particularly in the Zn-N, C-N, and SO_2_^−^ vibrational regions. Notably, the macrocyclic phthalocyanine ring exhibits characteristic C=N stretching vibrations around 1500–1600 cm^−1^, consistent with previous studies [39]. These vibrations not only confirm the aromatic character of the phthalocyanine core but also reflect its structural stability and integrity [40,41]. The absence of imaginary frequencies in the vibrational modes indicated that the optimized molecular structure was dynamically stable, further validating the computational approach. The observed vibrational features confirmed the structural integrity of Zn(SO_2_^−^)_4_Pc, making it suitable for applications in photocatalysis, sensors, and electronic devices. The strong Zn-N vibrations suggest a rigid coordination environment, which is essential for stability in catalytic and electrochemical processes. In addition, the distinct SO_2_^−^ vibrational signatures indicate enhanced solubility and functionalization potential, making this molecule a promising candidate for environmental remediation applications.

### 3.8. Cross-Correlation Between DFT Predictions and Membrane Properties

The integration of DFT and TD-DFT simulations provides critical insight into how molecular-level modifications influence bulk membrane properties. In particular, the introduction of Zn(SO_2_^−^)_4_Pc to the MPD system modifies the electronic and structural behavior of the resulting polyamide layer, which correlates directly with improvements in key membrane characteristics:

#### 3.8.1. Porosity (ε)

According to Han et al. (2025) [42], a higher molecular dipole moment enhances the interactions between polymers and solvents, often leading to looser chain packing. This looser packing increases the free volume within the polymer matrix, which in turn enhances membrane porosity.

The MPD/Zn(SO_2_^−^)_4_Pc complex in water displays a significantly higher dipole moment (20.30 D) than its individual components (approximately 2.6 D) (see Table 1). This substantial increase suggests strong charge separation and enhanced polarity, which facilitates favorable interactions with water molecules during interfacial polymerization. These strong dipole–solvent interactions can disrupt tight chain packing, promoting greater free volume generation and thereby increasing the porosity of the resulting polyamide membrane. Consequently, the elevated dipole moment of the complex in water supports the expectation of improved porosity, contributing to the enhanced water permeability and transport in the final membrane structure. The optimized geometries of the MPD/Zn (SO_2_^−^)_4_Pc complex show a more planar and extended conjugation system, which can promote better packing and structural organization during interfacial polymerization. This can lead to an increase in free volume and porosity within the membrane matrix, enhancing water flux. Porosity is defined as follows:

(2) $$\varepsilon =\left(1-\frac{\rho_{m}}{\rho_{p}}\right)\times 100$$

where

$\rho_{m}$ is the bulk density of the membrane.
$\rho_{p}$ is the polymer density.

#### 3.8.2. Hydrophilicity Mechanism

Molecular Electrostatic Potential (MEP) analysis revealed enriched regions of negative potential on sulfonate groups in Zn(SO_2_^−^)_4_Pc, indicating strong affinity for water molecules. These electronegative domains support enhanced hydrogen bonding and surface hydration, which translates to improved hydrophilicity and lower water contact angle (θ):

(3) $$Cos\theta =\frac{\gamma_{SV}-\gamma_{SL}}{\gamma_{LV}}$$

where

γ_SV_ is the solid–vapor interfacial tension.
γ_SL_ is the solid–liquid interfacial tension.
γ_LV_ is the liquid–vapor interfacial tension.

Increasing the surface polarity reduces the surface free energy (γ_SL_), which in turn enhances wettability and leads to a lower contact angle (θ). These computational results align with those of Politzer et al., who showed that areas with a high negative electrostatic potential are linked to stronger interactions with water and lower contact angles. This confirms that surface polarity is an important factor for improving hydrophilicity [43].

#### 3.8.3. Surface Roughness and π–π Interactions

The frontier molecular orbital of Zn(SO_2_^−^)_4_Pc analysis indicates significant delocalization in its HOMO of Zn(SO_2_^−^)_4_Pc, which enhances π–π stacking interactions with the aromatic rings of MPD. This interaction improves the molecular organization during membrane formation and affects the membrane surface roughness (Rq), an important parameter for antifouling behavior. The surface roughness was quantified as follows:

(4) $$R_{q}=\sqrt{\frac{1}{N}}\sum_{i=1}^{N}(z_{i}-\bar{z}{)}^{2}$$

where z_i_ is the height at each measured point and $\bar{z}$ is the mean surface height. R_q_ serves a dual purpose, depending on the hydrophilicity of the membrane. Higher R_q_ values lead to better antifouling properties by reducing the contact area for microbial adhesion. In hydrophilic systems involving MPD/Zn(SO_2_^−^)_4_Pc-modified membranes, increased surface roughness can enhance the stability of the hydration layer, thereby minimizing microbial adhesion and improving the antifouling performance [44]. This finding supports prior research showing that π–π stacking and aromatic conjugation can affect the nanostructure and void distribution within the polyamide layer, which in turn influences both water permeability and fouling resistance [45,46].

#### 3.8.4. Electronic Properties and Reactivity

The HOMO–LUMO gap (ΔE), a fundamental output of Density Functional Theory (DFT), provides critical insights into the electronic reactivity and crosslinking potential of membrane-forming monomers and additives [47,48]. A lower ΔE typically implies higher chemical reactivity because less energy is required to promote electron excitation [49]. In the context of interfacial polymerization, this translates into enhanced electron mobility and greater crosslinking efficiency within the polyamide layer.

As summarized in Table 2, the MPD molecule displayed a wide HOMO–LUMO gap of 5.61 eV, indicating high stability and low reactivity. In contrast, Zn(SO_2_^−^)_4_Pc shows a substantially lower gap of 1.32 eV, highlighting its favorable electronic properties. Upon complex formation (MPD/Zn(SO_2_^−^)_4_Pc), the gap in the gas phase decreases further to 1.23 eV in the gas phase, suggesting increased electron delocalization and a more reactive state conducive to integration during polymerization. Interestingly, when the complex was solvated in water, the gap increased slightly to 1.90 eV, which is consistent with the literature describing solvent-induced stabilization of charge-separated states (Owen et al., 2024) [50]. This moderate gap still supports electron participation in interfacial reactions, but with greater stability, an ideal balance for controlled membrane formation. These findings reinforce the utility of HOMO–LUMO gap analysis as a predictive tool for membrane-forming reactivity and solvent-dependent electronic behavior. The MPD/Zn(SO_2_^−^)_4_Pc complex demonstrated both enhanced reactivity and solvent-stabilized electronic properties, supporting its role in forming robust crosslinked polyamide membranes.

## 4. Conclusions

This study provides a comprehensive computational and spectroscopic analysis of Zn(SO_2_^−^)_4_Pc and its interaction with MPD, employing Density Functional Theory (DFT) and Time-Dependent DFT (TD-DFT) methodologies. The structural optimization of Zn (SO_2_^−^)_4_Pc and MPD/Zn(SO_2_^−^)_4_Pc confirmed their stability and electronic properties, with solvation effects playing a key role in stabilizing the MPD/Zn(SO_2_^−^)_4_Pc complex. The HOMO-LUMO energy gap analysis demonstrated a significant reduction in bandgap upon MPD functionalization, enhancing charge delocalization and improving optical and electronic properties. In contrast, the solvent phase (water) increased the bandgap, suggesting reduced charge transfer efficiency due to solvent polarization effects.

The UV–Vis spectral analysis, computed via TD-DFT, revealed a strong Q-band absorption at 611.417 nm, characteristic of π → π transitions in phthalocyanine systems*. The presence of sulfonate (-SO_2_^−^) groups contributed to spectral broadening and minor shifts, confirming their influence on electronic structure and solubility. The FT-IR vibrational analysis performed using RB3LYP/6-31G(d) identified Zn-N, C-N, and SO_2_^−^ stretching modes, which validate the structural integrity and dynamic stability of Zn(SO_2_^−^)_4_Pc. The Molecular Electrostatic Potential (MEP) analysis highlighted strong charge separation in Zn(SO_2_^−^)_4_Pc, with highly negative sulfonate groups and a positively charged Zn^2+^ core, making it a promising candidate for charge transfer interactions and catalytic applications. The MPD-functionalized system exhibited a more homogeneous charge distribution, indicating greater stability and enhanced charge delocalization, while solvent interactions further stabilized the electronic structure by shielding extreme charge separations.

To further interpret the structural behavior and guide molecular design, conformational analysis reveals that the MPD–Pc dihedral angle significantly influences molecular stability and electronic properties. The 120° conformation is preferred due to the synergistic effects of π–π stacking, hydrogen bonding, and minimal steric hindrance.

This research provides valuable insights into the structural and electronic behavior of Zn(SO_2_^−^)_4_Pc and MPD/Zn(SO_2_^−^)_4_Pc, supporting their potential use in membrane technology, optoelectronic applications, and charge transport systems. The findings particularly support the development of modified ultrafiltration (UF) and reverse osmosis (RO) membranes through interfacial polymerization, incorporating Zn(SO_2_^−^)_4_Pc with MPD in the non-solvent phase. The DFT and TD-DFT calculations confirm that the interactions between MPD in the aqueous phase and the organic phase containing TMC play a crucial role in optimizing polyamide membranes, influencing membrane porosity, stability, and dynamic performance. These results pave the way for next-generation membrane materials with improved stability, selectivity, and efficiency in water purification, pollutant removal, and gas separation applications. Future studies should focus on experimental validation and advanced solvent modeling to further refine the applicability of MPD/Zn (SO_2_^−^)_4_Pc-functionalized membranes in real-world filtration and separation technologies.

## Figures and Tables

**Table 1:** Description of minimum total energy and their dipole for MPD, Zn(SO_2_^−^)_4_Pc and MPD/Zn(SO_2_^−^)_4_Pc.

State	Phase	Minimum Total Energy (Hartree)	Dipole (Debye)
MPD	Gas phase	−342.954877	2.603122
Zn(SO_2_^−^)_4_Pc	Gas phase	−5638.225264	2.554394
MPD/Zn(SO_2_^−^)_4_Pc	Gas phase	−5981.218660	19.817231
MPD/Zn (SO_2_^−^)_4_Pc	In water	−5981.823923	23.18453

**Table 2:** Dihedral angle dependence of energy and dipole moment in MPD/Zn(SO_2_^−^)_4_Pc.

Dihedral Angle	Energy (Hartree)	Dipole Moment (Debye)
60°	−5981.218297	18.23
90°	−5981.214242	14.79
120°	−5981.218660	19.82
180°	−5981.218660	19.26

**Table 3:** Effect of dihedral angle on HOMO–LUMO energies and band gap of MPD/Zn(SO_2_^−^)_4_Pc complex (in eV).

Dihedral Angle	HOMO (eV)	LUMO (eV)	ΔE (eV)
60°	3.19625	−4.421036158	1.224240886
90°	3.21856	−4.427022666	1.208458274
120°	3.19326	−4.416138106	1.222880316
180°	3.20605	−4.440628366	1.234581218

**Table 4:** Energy gap ΔE_g_ comparison between MPD, Zn (SO_2_^−^)_4_Pc and MPD/Zn (SO_2_^−^)_4_Pc.

Molecule/System	Phase	ΔEg (eV)
(a) MPD	Gas phase	5.61 eV
(b) Zn(SO_2_^−^)_4_Pc	Gas phase	1.32 eV
(c) MPD/Zn(SO_2_^−^)_4_Pc	Gas phase	1.22eV
(d) MPD/Zn(SO_2_^−^)_4_Pc	In solvent water	1.89 eV

**Table 5:** Calculated global chemical reactivity descriptors [36].

Molecules	MPD	Zn(SO_2_^−^)_4_Pc	MPD/Zn(SO_2_^−^)_4_Pc (Gas Phase)	MPD/Zn(SO_2_^−^)_4_Pc (Water)
Ionization potential (IP = −E_HOMO_)	0.18457	−0.12296	−0.11735	0.16822
Electron affinity (EA = −E_LUMO_)	−0.02158	−0.17139	−0.16229	0.09879
Chemical hardness (η = (IP − EA)/2)	0.103075	0.02421	0.02247	0.03471
Chemical softness (s = 1/2η)	4.850836	20.64835	22.25189	14.40299
Chemical potential (μ = (IP + EA)/2)	−0.081495	−0.147175	−0.13982	−0.13350
Electronegativity (χ = (1 + EA)/2)	0.48921	0.147175	0.51123	0.51735
Electrophilicity index (ω = μ^2^/2η)	0.032216	0.4472533	0.435016	0.25671

## Data Availability

The data used to support the findings of this study are available from the corresponding authors upon request.

## Abbreviations

The following abbreviations are used in this manuscript:

MPD	Meta-phenylenediamine
Zn(SO2−)4Pc	Sulfonated zinc phthalocyanine
IPol	Interfacial polymerization
MEP	Molecular electrostatic potential
DFT	Density Functional Theory
TD-DFT	Time-Dependent DFT
HOMO	Highest occupied molecular orbital
LUMO	Lowest unoccupied molecular orbital

## References

[1] Shen S.L., Wang Y., Wang J.X., Li Y.H., Zhang J.F., Chen L. (2022). Fabrication of Thin-Film Composite Membranes with High Perm selectivity via Interfacial Polymerization. J. Membr. Sci..

[2] Tang L., Guo F., He L., Zhao H., Yang W., Wang X. (2021). Advances in Thin-Film Composite Membrane Design for Water Treatment. Desalination.

[3] Li Z., Ding H.J., Yang Y., Sun L., Xu R., Chen Y. (2023). Mitigating Chlorine Degradation in RO Membranes: A Comprehensive Review. J. Water Process Eng..

[4] Rahimpour A., Jahanshahi M., Rajaeian S.M., Tien H.N., Matsuura T., Ismail A.F. (2020). Nanomaterial-Based Modifications of Polyamide Membranes for Enhanced Performance. J. Membr. Sci..

[5] Szekeres M., Arthanareeswaran K., Asatekin J., Chen C., Gupta S. (2023). Phthalocyanine-Based Membranes for Water Purification and Selective Separation. J. Appl. Polym. Sci..

[6] Bottari G., Herranz M.Á., Wibmer L., Volland L., Laura Rodríguez-Pérez L., Guldi D.M., Hirsch A., Torres T. (2017). Chemical functionalization and characterization of graphene-based materials. Chem. Soc. Rev..

[7] Şahin G., Harmandar K., Jamoussi B., Durmuş M. (2020). The new water-soluble zinc (II) phthalocyanines substituted with morpholine groups: Synthesis and optical properties. J. Photochem. Photobiol. A Chem..

[8] Urbani M., Grätzel M., Nazeeruddin M.K., Martín N., D’Souza F., Torres T. (2014). Molecular Engineering of Phthalocyanines for Dye-Sensitized Solar Cells. Chem. Rev..

[9] Shabatina T.A., Vernigor A.A., Borisova N.A., Konovalov A.I. (2019). Coordination Chemistry of Phthalocyanines: Functionalization Strategies and Applications. Coord. Chem. Rev..

[10] Jiang J., Wang S., Lin J., Zhang W., Chen H., Liu Z. (2021). Tuning the Optoelectronic Properties of Phthalocyanines for Organic Electronic Devices. Adv. Funct. Mater..

[11] Fu X., Lin J., Liang Z., Yao R., Wu W., Fang Z., Zou W., Wu Z., Ning H., Peng J. (2023). Graphene oxide as a promising nanofiller for polymer composite. Surf. Interfaces.

[12] Tawfik A., Eraky M., Khalil M.N., Osman A.I., Rooney D.W. (2022). Sulfonated graphene nanomaterials for membrane antifouling, pollutant removal, and production of chemicals from biomass: A review. Environ. Chem. Lett..

[13] Rath R., Mohanty S., Kumar P., Nayak S.K., Unnikrishnan L. (2023). Synergistic effect of silica-covered graphene oxide (In-Situ) hybrid nanocomposites for use as a polymer electrolyte membrane for fuel cell applications. Surf. Interfaces.

[14] Ma L., Zhao D., Zheng J. (2019). Construction of electrostatic and π–π interaction to enhance interfacial adhesion between carbon nanoparticles and polymer matrix. J. Appl. Polym. Sci..

[15] Liu Y., Xiong H., Yu X., Huang H., Li L., Ji J., Huang Y., Xu M. (2017). Interfacial fabrication of polypyrrole/sulfonated reduced graphene oxide nanocomposites for electrochemical capacitors. Polym. Compos..

[16] Sun Y., Tian L., Qiao Z., Geng C., Guo X., Zhong C. (2022). Surface modification of bilayer structure on metal-organic frameworks towards mixed matrix membranes for efficient propylene/propane separation. J. Membr. Sci..

[17] Zhao Y., Chen Z., Li W., Huang B., Wang J., Sun P. (2022). DFT Study on Polyamide Membrane Functionalization for Improved Water Filtration. Theor. Chem. Acc..

[18] Hanack M., Schneider T., Eastwood D.E. (1998). Electronic Properties of Metal Phthalocyanines: A Computational Perspective. J. Porphyr. Phthalocyanines.

[19] Thomas K.R., Lin J.T., Hsu Y.C., Ho K.C. (2005). Organic dyes containing thienylfluorene conjugation for solar cells. Chem. Commun..

[20] Odobel F., Blart E., Lagree M., Pleux C. (2010). Based Photosensitizers: Applications in Light-Harvesting and Solar Energy Conversion. Coord. Chem. Rev..

[21] Jamoussi B., Al-Sharif M.N.M., Gzara L., Organji H., Almeelbi T.B., Chakroun R., Al-Mur B.A., Al Makishah N.H.M., Madkour M.H.F., Aloufi F.A. (2024). Hybrid Zinc Phthalocyanine/PVDF-HFP System for Reducing Biofouling in Water Desalination: DFT Theoretical and MolDock Investigations. Polymers.

[22] Sasa N., Okada K., Nakamura K., Okada S. (1998). Synthesis, structural and conformational analysis and chemical properties of phthalocyaninatometal complexes. J. Mol. Struct..

[23] Gajda Ł., Kupka T., Broda M.A. (2017). Solvent impact on the planarity and aromaticity of free and monohydrated zinc phthalocyanine: A theoretical study. Struct. Chem..

[24] Dennington R., Keith T.A., Millam J. (2016). GaussView.

[25] Frisch M.J., Trucks G.W., Schlegel H.B., Scuseria G.E., Robb M.A., Cheeseman J.R., Scalmani G., Barone V., Mennucci B., Petersson G.A. (2009). Gaussian 09.

[26] Solğun D.G., Yıldıko Ü., Ağırtaş M.S. (2021). Synthesis, DFT calculations, photophysical, photochemical properties of peripherally metallophthalocyanines bearing (2-(benzo[d] [1,3] dioxol-5-ylmethoxy) phenoxy) substituents. Research Square.

[27] Csonka G.I., French A.D., Johnson G.P. (2021). Density Functional Theory: A Powerful Tool for Predicting and Interpreting Vibrational Spectra. Molecules.

[28] Laurent A.D., Jacquemin D. (2013). TD-DFT benchmarks: A review. Int. J. Quantum Chem..

[29] Scheiner S. (2023). Molecular Electrostatic Potential Topology Analysis of Noncovalent Interactions. Acc. Chem. Res..

[30] Pechlaner M., van Gunsteren W.F. (2020). Algorithms to apply dihedral-angle constraints in molecular or stochastic dynamics simulations. J. Chem. Phys..

[31] Martins J.B.L., Cabral B.J.C. (2023). Electron binding energies of SO2 at the surface of a water cluster. J. Chem. Phys..

[32] Tomasi J., Mennucci B., Cammi R. (2005). Quantum Mechanical Continuum Solvation Models. Chem. Rev..

[33] Pearson R.G. (1989). Absolute electronegativity and hardness: Applications to organic chemistry. J. Org. Chem..

[34] Geerlings P., Proft F.D., Langenaeker W. (2003). Conceptual density functional theory. Chem. Rev..

[35] Padmanabhan J., Parthasarathi R., Subramanian V., Chattaraj P.K. (2007). Electrophilicity-based charge transfer descriptor. J. Phys. Chem..

[36] Villemin D., Abbaz T., Bendjeddou A. (2018). Molecular structure, HOMO, LUMO, MEP, natural bond orbital analysis of benzo and anthraquinodimethane derivatives. Pharm. Biol. Eval..

[37] Basha F., Khan F.L., Muthu S., Raja M. (2021). Computational evaluation on molecular structure (Monomer, Dimer), RDG, ELF, electronic (HOMO-LUMO, MEP) properties, and spectroscopic profiling of 8-Quinolinesulfonamide with molecular docking studies. Comput. Theor. Chem..

[38] Palewska K., Sworakowski J., Lipiński J., Nešpůrek S. (2011). Effect of electric permittivity of the solvent on aggregation process of the water-soluble sulfonated metal phthalocyanines. J. Photochem. Photobiol. A Chem..

[39] Xia D., Li W., Wang H., Zheng X., Guo Y., Yang X. (2011). Study on Spectra Properties of Novel Octa-Substituted Phthalocyanines. Spectrosc. Spectr. Anal..

[40] Tackley D.R., Dent G., Ewen Smith W. (2001). Phthalocyanines: Structure and vibrations. Phys. Chem. Chem. Phys..

[41] Napier A., Collins R.A. (1994). FTIR characteristics of halogenated phthalocyanines exhibiting polymorphism. Thin Solid Film.

[42] Han L., Fan C., Liu Y., Yang Y., Li H., Tang L., Li H., Liu Y., Wu H., Jiang Z. (2025). Engineering Solute–Solvent Interactions for the Synthesis of Covalent Organic Polymer Nanosheets. Small.

[43] Politzer P., Lane P., Murray J.S. (2016). Electrostatic Potentials, Intralattice Attractive Forces and Crystal Densities of Nitrogen-Rich C,H,N,O Salts. Crystals.

[44] Zhao D., Chen L., Peng M., Xue B., Yao Z., Huang W., Wang Z., Liu J. (2025). The complex influence of membrane roughness on colloidal fouling: A dialectical perspective. J. Membr. Sci..

[45] Ma X., Yang Z., Yao Z., Guo H., Xu Z., Tang C.Y. (2019). Tuning roughness features of thin film composite polyamide membranes for simultaneously enhanced permeability, selectivity and anti-fouling performance. J. Colloid. Interface Sci..

[46] Bhalani D.V., Trivedi J.S., Jewrajka S.K. (2021). Selective grafting of morphologically modified poly(vinylidene fluoride) ultrafiltration membrane by poly(acrylic acid) for inducing antifouling property. Appl. Surf. Sci..

[47] Yankova R., Yotova T.S., Avramov M. (2025). DFT Investigation of Electronic Structure, Reactivity, and Molecular Interactions in a Selenate–Selenite System. Russ. J. Gen. Chem..

[48] Qi Y., Gong W., Yan Q. (2025). Bridging deep learning force fields and electronic structures with a physics-informed approach. Npj Comput. Mater..

[49] Chahmana S., Benghanem F., Fellah M., Aityoucef H., Bennaadja S., Foudia M., Djili A., Ghedjati S., El-Hiti G.A. (2024). Integrated exploration of molecular structure, quantum chemical properties, molecular docking, and antioxidant activity of 4-(2-hydroxyanilino)pent-3-en-2-one. Results Chem..

[50] Owen A.E., Anyambula I.A., Benson C.U., Ojumola F.O., Alawa J.A., Benjamin I., Iyam S.O., Ogar C.U., Ojong M.A., Ojong R. (2024). Exploration of semi-carbazone derivatives as promising agents against cholera: Insights from spectroscopic analysis, reactivity studies (ELF, HOMO-LUMO, NBO), solvation effects, and molecular docking investigations. Chem. Phys. Impact.

